# Mogens Lesner Glass (1946-2018)

**DOI:** 10.1590/1414-431X202010838

**Published:** 2020-11-18

**Authors:** Tobias Wang, Steve Wood

**Affiliations:** 1 Section for Zoophysiology, Department of Bioscience, Aarhus University, C.F. Møllers Allé 3, Aarhus, 8000 Aarhus C, Denmark; 2 102 Skyland Blvd., Tijeras, NM 87059, USA

**Figure:**
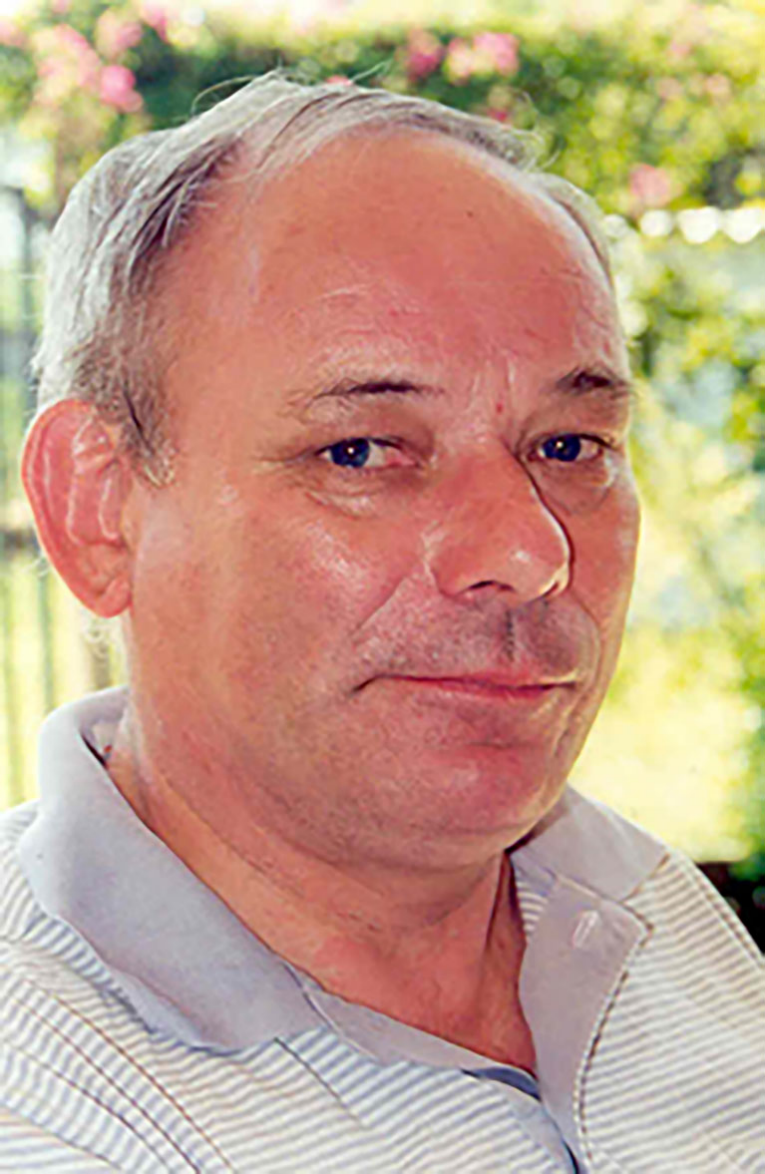


On October 4, 2018, the professor, doctor, and scientist Mogens Lesner Glass died at his home in Ribeirão Preto, Southeastern Brazil, after a prolonged illness. Mogens had retired a few years earlier from a full professor position in the Department of Physiology at Universidade de São Paulo in Ribeirão Preto. He provided important contributions to comparative respiratory physiology and trained a number of excellent students in Brazil, many of whom are now leading researchers at various universities in Southeastern Brazil. His role as a mentor and his work has, and will continue to have, a lasting impact on the study of comparative physiology in Brazil. (Figure 1)

## Childhood and becoming a biologist

Mogens was born in Aarhus, the second largest city in Denmark, on March 24, 1946 and attended a private school (Forældreskolen) and finished high school nearby, in 1965. In high school (Marselisborg Gymnasium), he followed the linguistic line and therefore received little education in mathematics and physics. Thus, after having spent a year at Askov Højskole, following a century-old Danish tradition of attending a boarding school for additional general education prior to university, he needed to take extra courses in mathematics to enter the biology program at Aarhus University. In these courses, he received much needed help from his younger sister Birte and would later emphasize the quantitative approach to respiratory physiology.

**Figure 2:**
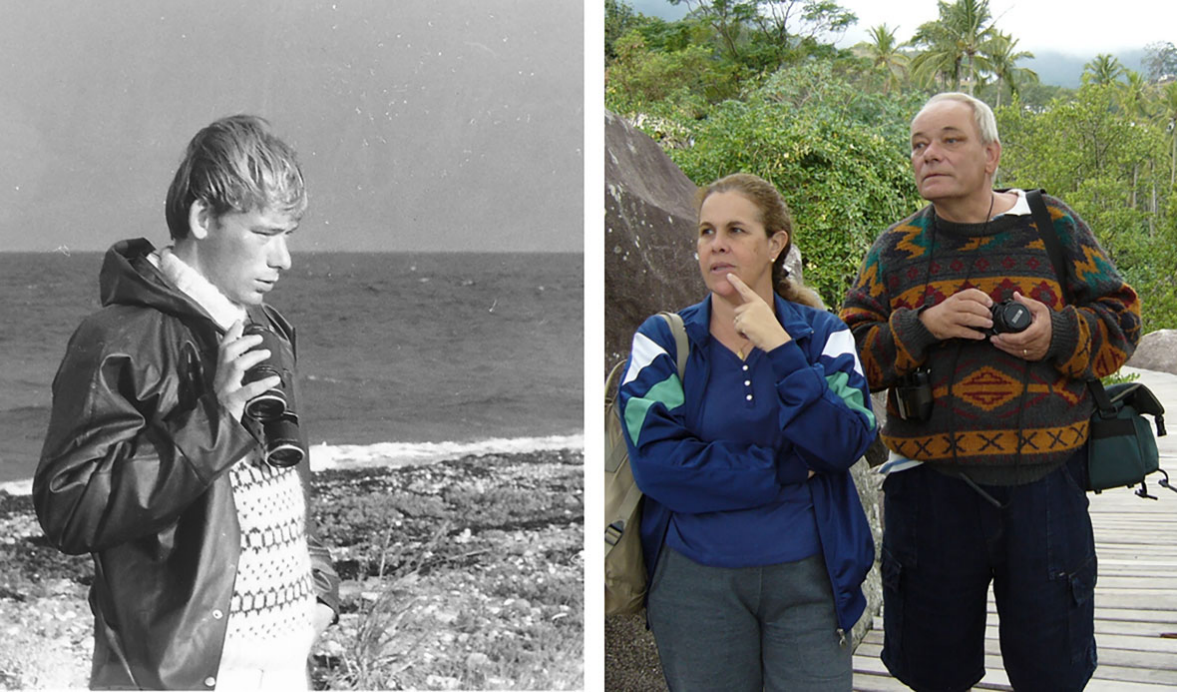
Mogens was a keen ornithologist throughout his life. On the left as a young university student and on the right with his wife Heloisa.

It came as no surprise that Mogens chose to study biology. Still in early childhood, he had developed a keen interest in natural history, and particularly loved birds; he remained a life-long amateur ornithologist and would treasure walks in nature throughout his life. As a school-boy, Mogens was active in the youth organization Nature and Youth (Natur og Ungdom). Later he enrolled in the newly established biology program at Aarhus University in 1966 and maintained his interest in ornithology, publishing two papers on the roost behavior of long-eared owls (*Asio otus*). In these two papers in the local, but well-respected, *Danish Journal for Ornithology*, Mogens described that the evening departure of the first owl from the roost could be explained by light intensity, and independent of weather conditions (Glass and Nielsen, 1967; Glass, 1971). During this time, he interacted with the entomologist Dr. Tetens Nielsen, a student of August Krogh, so he was in good academic company from the early start of his career. (Figure 2)

When Mogens started at Aarhus University, the new Institute of Biology was still in a formative process as the first students enrolled, and many of the more specialized courses had to be taken at the University of Copenhagen. Mogens thus relocated to the Danish capital for a few years, but returned to Aarhus for his MSc degree in zoophysiology (the Scandinavian name for comparative physiology) to be supervised by the Norwegian-born Viking and Physiologist Kjell Johansen (1932-1987).

Johansen had been lured to Aarhus from a tenured position at the University of Washington. He was given plenty of space for laboratories, offices, large climatic chambers, financial support to purchase state-of-the-art equipment, and was allowed to establish an animal care facility in the basement to maintain exotic animals that were imported from all over the world. Mogens was the second MSc student to graduate from the new Department of Zoophysiology.

## Master’s degree in zoophysiology

By spending his “formative years” under the inspiring tutelage of Professor Johansen, Mogens was immersed in an internationally-oriented research environment with many visitors, excellent facilities, and enormous enthusiasm (e.g. Johansen, 1987). This was at the end of the ‘golden era of comparative physiology’ (1960-1980), where many basic physiological functions were being described in non-mammalian vertebrate classes and the general physiological principles applicable to all species emerged and were defined.

**Figure 3:**
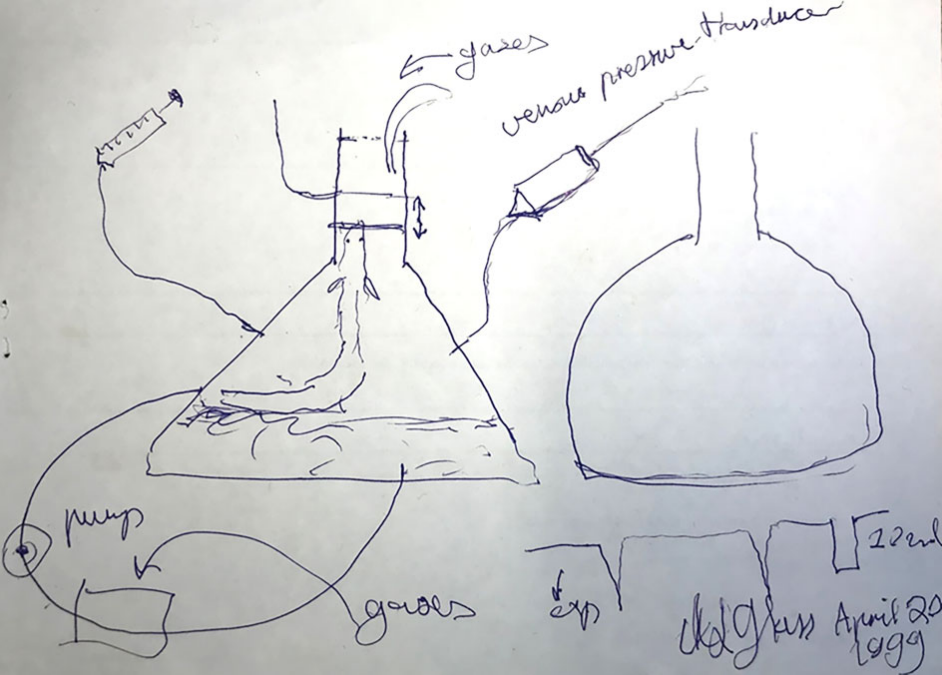
A “back-of-the-envelope” drawing by Mogens Glass from April 20, 1999 on Avenida do Café in Ribeirão Preto, Brazil. The figure depicts the set-up used to measure ventilation in aquatic snakes for his Master’s thesis. Here it is used for lungfish in a study on ventilatory responses to hypoxia (Sanchez et al., 2001).

Mogens’ MSc thesis concerned the regulation of lung ventilation and the breathing pattern of the aquatic snake *Acrochordus javanicus*, and the findings were already published by the time of the oral thesis defense on January 24, 1977 (Glass and Johansen, 1976). This publication still stands as one of the first and definitive studies of ventilatory responses to CO_2_ and low oxygen in unrestrained animals. Mogens also described how the breathing pattern and the small lungs enabled these snakes to remain submerged for more than 90% of the time without compromising buoyancy. (Figure 3)

Johansen’s integrative view and emphasis on studying the entire organism, under the view that the whole is greater than the sum of its parts, would have a lasting impact on Mogens’ research philosophy. Johansen also introduced Mogens to the academic and practical benefits of collaborating with medical schools. For example, Mogens enjoyed telling the story in which he and Johansen transported a large crocodile to the university hospital to take x-rays of its heart. Much to the horror of the patients waiting in the lobby, the tail became visible under the cloth covering the crocodile and, as commotion was about to ensue, Johansen calmly advised Mogens to “keep going at a steady pace”. What became of the x-rays was rarely included in the story, but every time he recounted it, Mogens would chuckle at great length in his characteristic manner.

## Visit to Albuquerque, New Mexico

**Figure 4:**
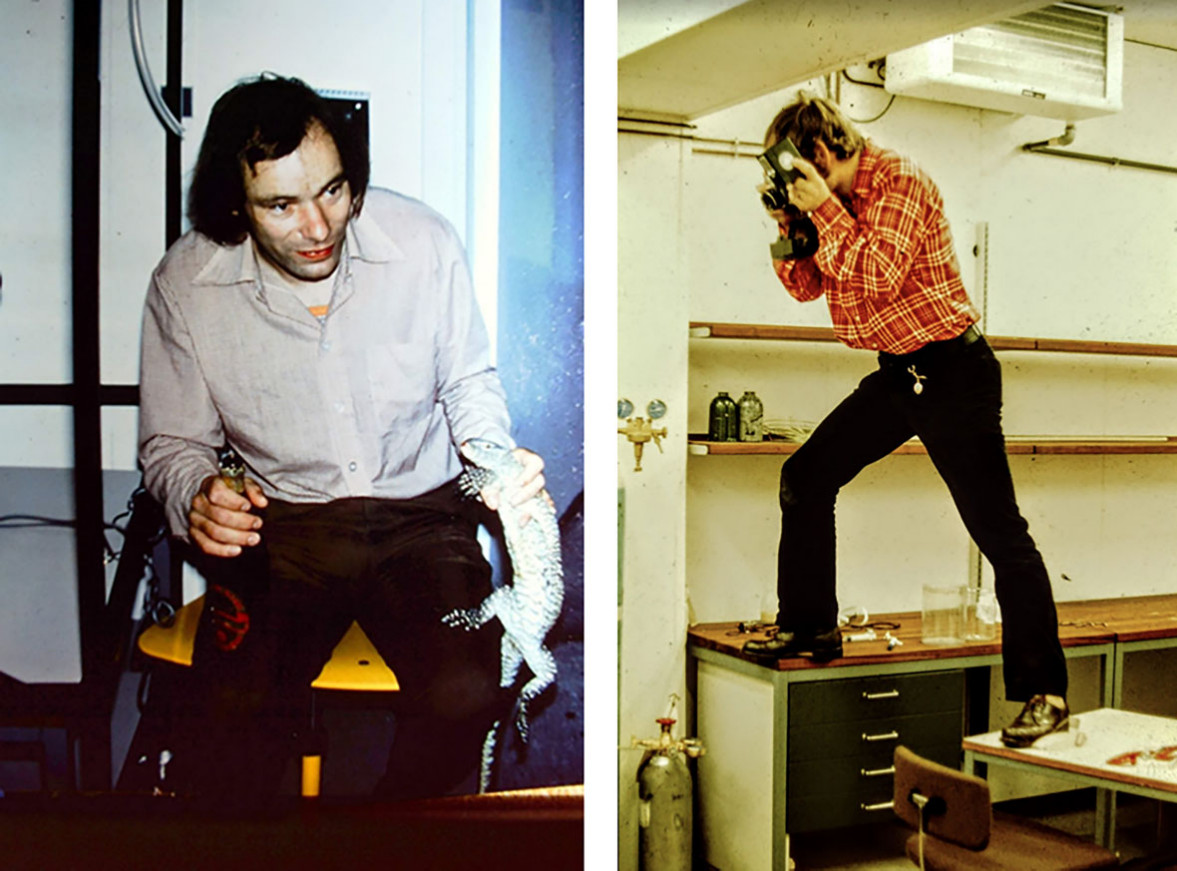
Mogens Glass with a Savannah monitor around 1981 (left panel) when he and collaborators demonstrated that this species of lizard (*Varanus exanthematicus*) does not reduce arterial pH with increased body temperature, providing strong evidence against the ubiquity of the α-stat hypothesis. In the right panel, Mogens in 1977 with one leg on a solid bench and the other on a table with wheels (you can guess the rest).

There was no formal PhD program at the Danish universities when Mogens had completed his MSc, so he enrolled as a so-called *licentiat scientarium* student with a university stipend that allowed him to continue research in respiratory physiology. Together with Dr. Steve Wood, who regularly visited Aarhus University during that period, Mogens developed a mask technique to measure ventilatory flow on unrestrained animals (Glass et al., 1978). This technique would be used in many of Mogens’ later studies. (Figure 4)

Mogens received support from Aarhus University to visit Steve Wood at University of New Mexico in Albuquerque in 1978, and he would later return to Albuquerque for a number of shorter trips. In New Mexico, Mogens traveled widely in his beloved jeep, he enjoyed cross-country skiing, and improved his already high proficiency in judo. He was a popular lecturer, giving many lectures on physiology to physical therapy students, and he collaborated with Marvin Riedesel and Jim Hicks on the effect of long-term acclimation to low temperatures on blood gases (Glass et al., 1979). During these experiments, Jim Hicks became the first of many students to have his clothes demolished by the two-component glue required to fasten the masks over the nostrils of the experimental animals.

## Alpha Helix expedition to study lung function of green sea turtles in Costa Rica

**Figure 5:**
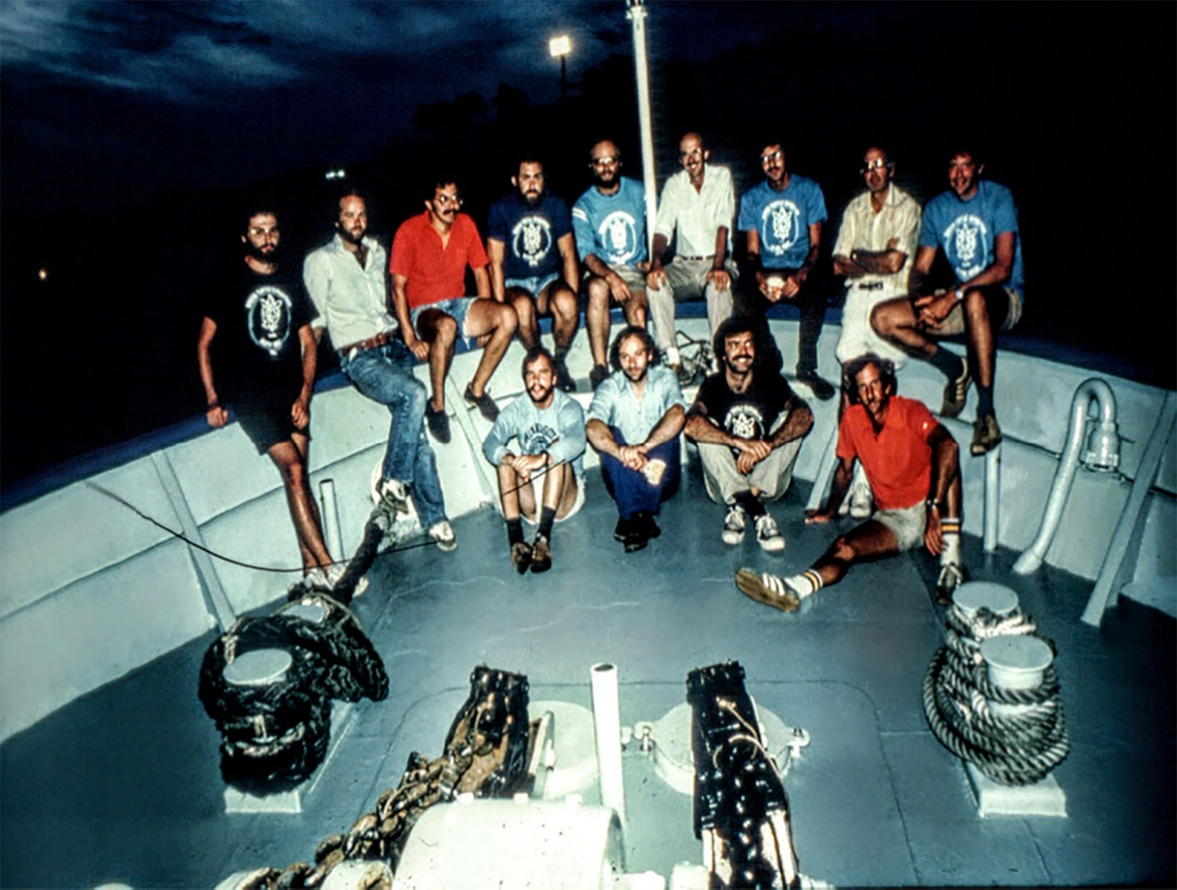
Mogens (front row center between Alan Hargens and Ron Millard) on the 1978 R/V Alpha Helix expedition to study green sea turtles in Puerto Limon, Costa Rica.

While in Albuquerque, Mogens was invited by Henry Prange and Donald C. Jackson to join the 1978 R/V Alpha Helix expedition to study green sea turtles in Puerto Limon, Costa Rica. As a collaborator with Steve Wood and Randy Gatz, Mogens measured the high blood oxygen binding affinity that likely allowed for high arterial oxygen saturation during prolonged dives (Wood et al., 1984). They also used the Fick principle to determine systemic blood flow, while pulmonary flow was measured by the rate of acetylene uptake. Pulmonary and systemic flows were very similar, which in combination with high arterial oxygen saturation provided evidence for very small cardiac shunts in conscious sea turtles. In a separate study on pulmonary function, the carbon-monoxide technique was applied to determine the diffusion capacity of the sea turtle lung (Gatz et al., 1987). (Figure 5)

Mogens maintained a long-lasting and close friendship with Steve Wood, and they published almost 20 papers together and edited several books (e.g. Glass and Wood, 2009). As an early and important result of their collaboration, Mogens and Steve were invited to review the existing knowledge on ventilatory control in reptiles for *Physiological Reviews* (Glass and Wood, 1983). In that paper, they provided strong evidence for ventilation and blood gases being exquisitely “well-regulated”, a view that was contrary to the earlier notion of ectotherms being “primitive” and rather incapable of precise regulation. Their seminal review would shortly thereafter form the introductory chapter to Mogens’ doctoral degree.

## Back in Aarhus to continue studies in comparative respiratory physiology

Upon returning to Aarhus in 1979, Mogens collaborated extensively with Warren Burggren, Augusto S. Abe, and Kjell Johansen to quantify the pulmonary diffusion capacity in a variety of amphibians and reptiles using the CO method. Mogens always enjoyed telling how this classic technique had been developed by Marie Krogh in Copenhagen already in 1915 (Krogh, 1915). Their studies demonstrated that the lower pulmonary diffusing capacity of ectothermic vertebrates is proportional to the lower rates of gas exchange compared with endotherms (e.g. Glass et al., 1981a,b). They also established that the increased pulmonary diffusion capacity for oxygen uptake with increases in temperature can be ascribed to a concomitant rise in pulmonary blood flow. Some of the resulting publications are still amongst the best studies on lung function in amphibians and reptiles, and were reviewed a decade later (Glass, 1991).

In Aarhus, Mogens also formed important friendships with Tadeu Rantin and Augusto Abe, both of whom independently visited Kjell Johansen as young postdoctoral fellows from Brazil. Mogens and Augusto collaborated on lung studies and ventilatory regulation in reptiles (Glass et al., 1981a,b; Kruhøffer et al., 1987). Mogens also became involved in studies of ventilatory regulation in air-breathing fish when Atsushi Ishimatsu visited Aarhus with boxes of snakehead fish (Glass et al., 1986). In all animals studied, the ventilatory responses were much more vigorous as temperature increased (e.g. Kruhøffer et al., 1987 [Bibr B30]).

To understand how and why body temperature influences arterial blood gases (PO_2_, PCO_2_, and pH), Mogens was particularly inspired by Steve Wood’s analyses of the influence of cardiac shunts (Wood, 1982, 1984). Indwelling catheters were placed in a number of species so arterial blood samples could be taken at various temperatures with minimal disturbance. Particularly, the finding that varanid lizards maintained constant arterial pH over a large range of body temperatures (Wood et al., 1977 and 1981) spurred Mogens to formulate a fierce criticism of the α-stat hypothesis that had been formulated by Robert Blake Reeves in 1972 (Reeves, 1972). Reeves proposed that ectothermic animals regulate protein ionization by reducing arterial pH with increased body temperature to match the effects of temperature on the dissociation constant for the α-imidazole group on histidines (see also Wang and Jackson, 2016). Mogens and collaborators, however, maintained that the reduction in arterial pH rarely matched the reduction in pK (e.g. Wood et al., 1977, 1987). In later studies, it was also argued that the ventilatory responses to pH in the cerebrospinal fluid surrounding the central chemoreceptors were inconsistent with the α-stat hypothesis (Branco et al., 1993).

## Postdoctoral studies at Max Planck Institute for Experimental Medicine in Göttingen

Despite the very impressive quality and quantity of Mogens’ scientific production, there was no prospect of a permanent position in Aarhus. Over a number of years, he alternated between short-time affiliations in Aarhus and the Max Planck Institute for Experimental Medicine in Göttingen where he became a very prolific research fellow.

**Figure 6:**
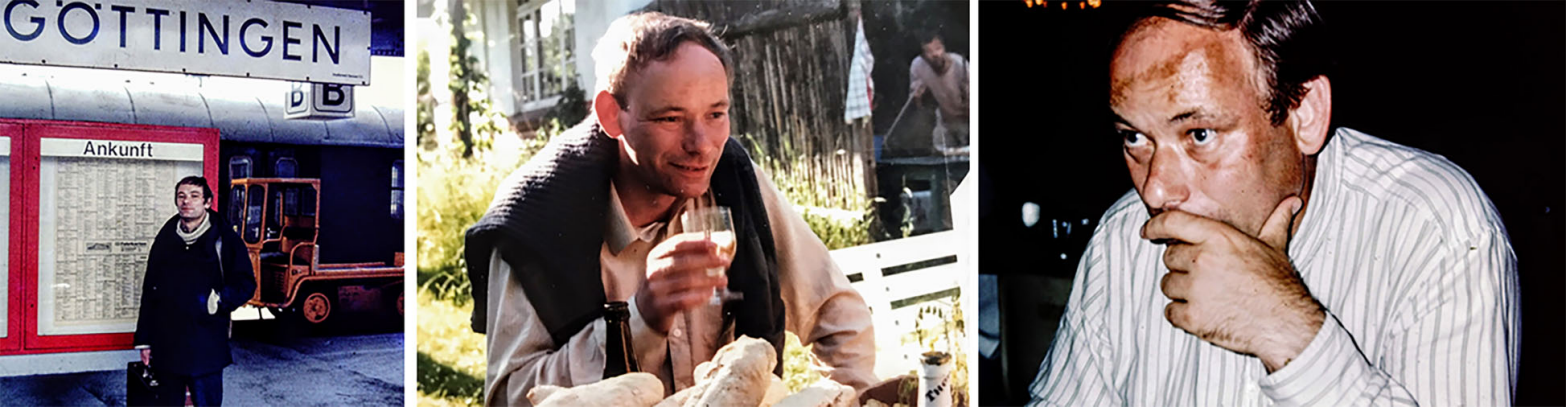
Mogens Glass in 1977 arriving at the train station in Göttingen for a research visit to the Max-Plank Institute (left), enjoying social company (center), and in his characteristic posture, thinking on how to enter the conversation with an opinion on how to understand a particular problem (right).

In Göttingen, Mogens published studies with Johannes Piiper, Peter Scheid, and Norbert Heisler as well as many of the international visitors. He was involved in a large number of different projects, but his collaboration with Bob Boutilier and Norbert Heisler on the relationship between the hypoxic ventilatory response and blood oxygen binding properties is probably amongst his most important contributions. In a seminal paper, they described how the rise in ventilation during hypoxia is matched to the arterial partial pressure of oxygen where blood oxygen concentration decreases markedly, *i.e.* the steep part of the oxygen equilibrium curve (Glass et al., 1983). This made perfect teleological sense because the ventilatory response matched blood oxygen delivery, and, as pointed out by the authors, raised the interesting question of whether the oxygen-sensitive chemoreceptors in reptiles respond to the partial pressure of oxygen or oxygen concentration. Subsequent studies with Luiz Guilherme Branco and Tobias Wang (both PhD students in Mogens lab in 1991) pointed to partial pressure as the regulated variable (Wang et al., 1994), but the debate continues to this day (Milsom and Wang, 2017). (Figure 6)

## Doctoral degree

In 1985, Mogens collated 12 publications from the years 1978 to 1984 into a doctoral thesis. Denmark has the doctoral degree (*doctor scientiarum*) as the highest academic degree, which is above a PhD degree in importance and must be based on individual unsupervised scholarly activities. True to form, Mogens went for this degree without bothering to complete the equivalent of a PhD thesis at the time. A picture of a crocodile and a trace of its intermittent ventilatory pattern appeared on the cover of his thesis entitled “*Respiration in Reptiles*”. In his own words, Mogens concluded that his studies “*describe flexible and complicated mechanisms for the gas exchange and blood gases in reptiles. The opinion that the control of breathing in reptiles is erratic may result from an incomplete understanding of the complicated regulatory patterns. Also, the cardiopulmonary functions of reptiles are adjusted to their low aerobic metabolism but function adequately to demands for oxygen and the need for acid-base balance*”. Professor Donald C. Jackson from Brown University (1938-2020), an eminent expert on the influence of temperature on blood gases and pulmonary ventilation in reptiles (Wang et al., 2020), served as the opponent at the oral defense.

During the years when Mogens moved frequently between Aarhus and Göttingen, he drove his beloved old Volvo accompanied by Thor, a charming dog brought back from New Mexico that would take Mogens on daily walks in the nearby forest; whether this impetuous and headstrong dog was named after the Nordic God of thunder or the strong Danish beer bearing the same name shall forever remain an unsolved mystery. One skill that Mogens decisively lacked was parallel parking, for which he invariably relied on what he called, “the acoustic method” to park between two other cars.

## Becoming full professor at Faculdade de Medicina in Ribeirão Preto

Amidst the many hours spent traveling and the lack of academic job security, forces in Brazil had been at work. Tadeu Rantin and Augusto Abe played an important role by suggesting Mogens to Jose Antunes Rodrigues, then head of the Physiology Department at Universidade de São Paulo (USP) in Ribeirão Preto, as a suitable candidate for a position in respiratory physiology that became available. As a result of these efforts, Mogens was offered a permanent job at one of the best medical schools in South America - Faculdade de Medicina in Ribeirão Preto, situated on a picturesque old coffee farm outside one of the largest cities in the interior of São Paulo.

Mogens arrived in Brazil in February 1989 and quickly established an active lab that pursued central questions in respiratory physiology, despite having rather limited resources. He arranged a large international conference in collaboration with Eduardo Bicudo at a wonderfully scenic location in San Sebastião, with the participation of many leading researchers that later would form lasting ties to comparative physiology in Brazil (see Bicudo (1992) for the entire conference issue).

Luiz Guilherme Branco, as the first PhD student under Mogens supervision, embarked on the first demonstration of central chemoreceptors playing a pivotal role for the regulation of ventilation in amphibians (Branco et al., 1992). The studies involved perfusion of the fourth ventricle with mock cerebrospinal fluid as the conscious toads were exposed to increasing levels of CO_2_ in the inspired air, a finding he subsequently expanded to lungfish (Amin-Navas, 2007). He also continued studies on other aspects of ventilatory control, branched further into studies of various tropical fishes, particularly in collaboration with Tadeu Rantin and Ana Kalinin in São Carlos, and he engaged in a number of collaborations with Professors Helio Salgado, Anete Hoffmann, and Benedito Machado on various cardiovascular research projects within the Physiology Department in Ribeirão Preto. He also collaborated widely with researchers at the clinical hospital located behind Faculdade de Medicina.

## Studies on respiratory physiology of lungfish

Having settled in Ribeirão Preto, Mogens turned his attention to the South American lungfish *Lepidosiren paradoxa*. Lungfish have their own class (Dipnoi) and form a sister group to the tetrapods (amphibians, reptiles, birds, and mammals), but they never colonized terrestrial environments. Their physiology is likely, therefore, to provide some insight into changes in respiratory gas exchange and its regulation during the transition from aquatic to terrestrial lifestyles. In the many studies on *Lepidosiren*, a quest that involved a large number of graduate students and the devoted technician Humberto Guisto, Mogens utilized all the techniques and approaches he had accumulated over the previous decades, and his laboratory therefore provided a very extensive description of the respiratory physiology and lung function of this rather unique species. Before retirement, Mogens reviewed some of the studies (Glass et al., 2007, 2008, 2009 [Bibr B19]), and Glauber da Silva, Mogens’ last student, recently provided a review with a number of collaborators (Nunan et al., 2019). The overall conclusion from the lungfish studies is that virtually all parameters of ventilatory regulation in lungfish resemble those of the tetrapods. Thus, the transitions in regulation took place prior to colonization of land and seem to be primarily associated with the transition to obligate air-breathing.

## Mogens as a mentor, colleague, and friend

**Figure 7:**
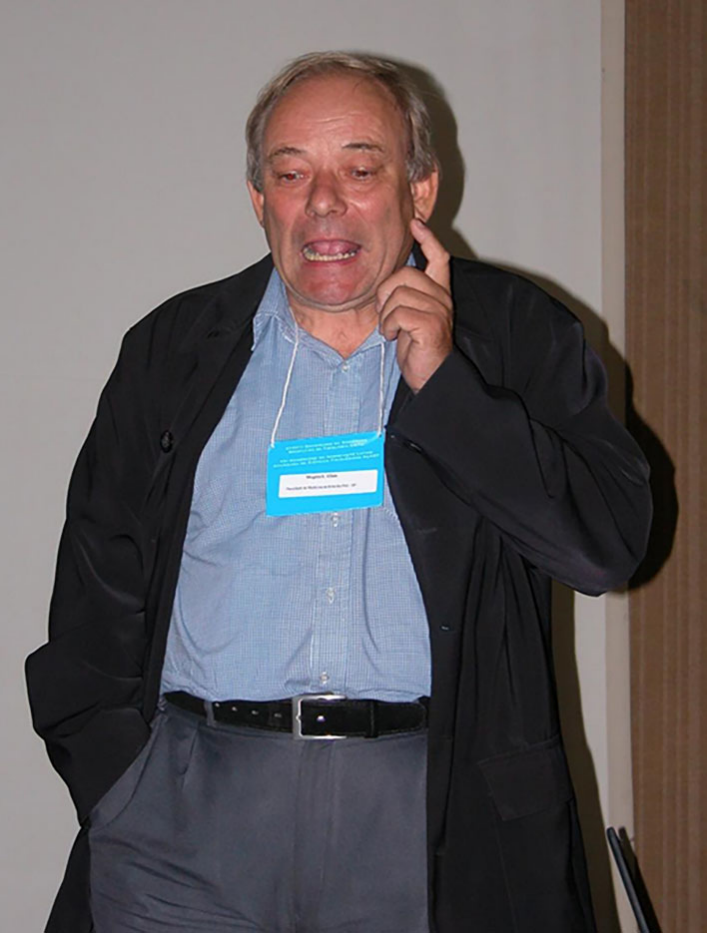
Mogens was a gifted speaker with the ability to deliver talks at a high academic level with many subtle jokes. Here, Mogens is answering questions after an invited talk at the yearly meeting of the Brazilian Physiological Society in Ribeirão Preto, Brazil, in 2003.

Mogens was a very unique person, and probably the most eccentric person we have known, but all people who became acquainted with him over the years cherished his characteristic mannerisms. For example, anyone who has ridden in the old Volvo with him will fondly remember his distinct manner of changing gears, and the odd little bell installed for mysterious purposes. Mogens was an extraordinarily generous human being and cared deeply for the people around him. He possessed a great sense of humor, an extraordinary knowledge of languages and history, as well as a love for music and arts. Mogens was extraordinarily entertaining company; he could entertain large groups of people, but typically preferred small social circles where he could talk about literature, classical music, history of sciences, and art. He was a devoted supervisor with immense patience, and enjoyed taking his protégées on long walks where he would tell stories of other scientists and explain his views on particular problems. He was immensely inspiring. Endowed with an uncanny ability to reduce complex problems to the basic relationships, he would - when given a pen and the back of an envelope - derive equations and develop ideas for experimental tests. His enthusiasm was particularly contagious in those situations. (Figure 7)

Mogens was a clever scientist bestowed with the rare ability of combining quantitative approaches, a good understanding of physical chemistry, an appreciation of natural history, and a great deal of common sense. He was a beloved friend to many of those he met in numerous countries. The world became a considerably less interesting place with the passing of Mogens and he will surely be missed by many friends on numerous continents. He is survived by his wife Virginia, his son Otto, and his younger sister Birte.

*Tobias Wang is the current head of the Section of Zoophysiology at Aarhus University and met Mogens for the first time in 1987. He worked with Mogens in 1989 when spending a semester with Augusto Abe at UNESP in Rio Claro as an undergraduate student. Tobias later returned to Rio Claro and Ribeirão Preto for a year in 1991 as a first-year PhD student and visited Mogens many times since then.*

*Steve Wood got acquainted with Mogens in Aarhus during the 1970s and they remained close friends until Mogens died. Steve Wood is now retired from his full-time position, but is still teaching a course in pathophysiology at the University of New Mexico Taos campus.*

## References

[B01] Amin-Naves J, Giusti H, Hoffmann A, Glass ML (2007). Central ventilatory control in the south American lungfish, *Lepidosiren paradoxa*: contributions of pH and CO_2_. J Comp Physiol.

[B02] Branco LGS, Glass ML, Hoffman A (1992). Central chemoreceptor drive to breathing in unanesthetized toads, Bufo paracnemis. Respir Physiol.

[B03] Branco LGS, Glass ML, Wang T, Hoffmann A (1993). Temperature and central chemoreceptor drive to ventilation in toad (*Bufo paracnemis*). Respir Physiol.

[B04] Gatz RN, Glass ML, Wood SC (1987). Pulmonary function of the green sea turtle, *Chelonia mydas*. J Appl Physiol.

[B05] Glass ML, Nielsen TH (1967). On the evening departure of the long-eared owl (*Asio otus*) from the winter roost. Dansk Ornitologisk Forenings Tidsskrift.

[B06] Glass ML (1971). Some remarks on the evening departure of the long-eared owl (*Asio otus*) from the winter roost. Dansk Ornitologisk Forenings Tidsskrift.

[B07] Glass ML, Johansen K (1976). Control of breathing in *Acrochordus javanicus*, an aquatic snake. Physiol Zool.

[B08] Glass ML, Wood SC, Johansen K (1978). The application of pneumotachography on small unrestrained animals. Comp Biochem Physiol.

[B09] Glass ML, Hicks JW, Riedesel ML (1979). Respiratory responses to long-term temperature exposure in the box turtle, *Terrapene ornate*. J Comp Physiol.

[B10] Glass ML, Abe AS, Johansen K (1981a;). Pulmonary diffusing capacity in reptiles: relations to temperature and O_2_ uptake. J Comp Physiol.

[B11] Glass ML, Burggren WW, Johansen K (1981b). Pulmonary diffusing capacity of the bullfrog (*Rana catesbeiana*). Acta Physiol Scand.

[B12] Glass ML, Wood SC (1983). Gas exchange and control of breathing in reptiles. Physiol Rev.

[B13] Glass ML, Boutilier RG, Heisler N (1983). Ventilatory control of arterial PO_2_ in the turtle *Chrysemys picta bellii*: Effects of temperature and hypoxia. J Comp Physiol.

[B14] Glass ML, Ìshimatsu A, Johansen K (1986). Responses of aerial ventilation to hypoxia and hypercapnia in *Channa argus*, and air-breathing fish. J Comp Physiol.

[B15] Glass ML, Wood SC (2009). Cardio-Respiratory Control in Vertebrates. Comparative and Evolutionary Aspects.

[B16] Glass ML, Bicudo JEPW (1991). Pulmonary diffusion capacity of ectothermic vertebrates. The Vertebrate Gas Transport Cascade.

[B17] Glass ML (2008). The enigma of aestivation in the African lungfish *Protopterus dolloi* - commentary on the paper by Perry et al. Respir Physiol Neurobiol.

[B18] Glass ML, Sanchez AP, Amin-Naves J, Bassi M, Rantin FT (2007). Respiratory Function in the South American Lungfish, *Lepidosiren paradoxa*. Fish Respiration and Environment.

[B19] Glass ML (2009). Physiological evidence indicates lungfish as a sister group to the land vertebrates. Cardio-Respiratory Control in Vertebrates Berlin.

[B20] Johansen K (1987). The August Krogh Lecture: the world as a laboratory - physiological insights from nature’s experiments. Advances in Physiological Research (McLennan H, Ledsome JR, McIntosh CHS, Jones DR, Editors), Plenum Press.

[B21] Krogh M (1915). The diffusion of gases through the lungs of man. J Physiol.

[B22] Kruhøffer M, Glass ML, Abe AS, Johansen K (1987). Control of breathing in an amphibian *Bufo paracnemis* - effects of temperature and hypoxia. Respir Physiol.

[B23] Milsom WK, Wang T (2017). Is the hypoxic ventilatory response driven by blood oxygen concentration?. J Exp Biol.

[B24] Reeves RB (1972). An imidazole alphastat hypothesis for vertebrate acid-base regulation: Tissue carbon dioxide content and body temperature in bullfrogs. Respir Physiol.

[B25] Sanchez A, Soncini R, Wang T, Koldkjær P, Taylor EW, Glass ML (2001). The differential cardio-respiratory responses to ambient hypoxia and systemic hypoxaemia in the South American lungfish, *Lepidosiren paradoxa*. Comp Biochem Physiol.

[B26] Wang T, Branco LGS, Glass ML (1994). Ventilatory responses to hypoxia in the toad *Bufo paracnemis* before and after a decrease in haemoglobin oxygen-carrying capacity. J Exp Biol.

[B27] Wang T, Jackson DC (2016). How and why pH changes with body temperature. J Exp Biol.

[B28] Wang T, Warren DE, Warburton S (2020). Donald C. Jackson (1937-2020). J Exp Biol.

[B29] Wood SC, Glass ML, Johansen K (1977). Effects of temperature on respiration and acid-base balance in a monitor lizard. J Comp Physiol.

[B30] Wood SC, Johansen K, Glass ML, Hoyt RW (1981). Acid-base regulation during heating and cooling in the lizard, *Varanus exanthematicus.*. J Appl Physiol.

[B31] Wood SC (1982). The effect of oxygen affinity on arterial PO_2_ in animals with vascular shunts. J Appl Physiol.

[B32] Wood SC (1984). Cardiovascular shunts and oxygen transport in lower vertebrates. Am J Physiol.

[B33] Wood SC, Gatz RN, Glass ML (1984). Oxygen transport in the green sea turtle. J Comp Physiol.

